# Liquid biopsy with multiplex ligation-dependent probe amplification targeting cell-free tumor DNA in cerebrospinal fluid from patients with adult diffuse glioma

**DOI:** 10.1093/noajnl/vdac178

**Published:** 2022-11-25

**Authors:** Ryosuke Otsuji, Yutaka Fujioka, Nobuhiro Hata, Daisuke Kuga, Yuhei Sangatsuda, Kosuke Takigawa, Yusuke Funakoshi, Aki Sako, Hidetaka Yamamoto, Akira Nakamizo, Masahiro Mizoguchi, Koji Yoshimoto

**Affiliations:** Department of Neurosurgery, Graduate School of Medical Sciences, Kyushu University, 3-1-1 Maidashi, Higashi-ku, Fukuoka 812-8582, Japan; Department of Neurosurgery, Graduate School of Medical Sciences, Kyushu University, 3-1-1 Maidashi, Higashi-ku, Fukuoka 812-8582, Japan; Department of Neurosurgery, Graduate School of Medical Sciences, Kyushu University, 3-1-1 Maidashi, Higashi-ku, Fukuoka 812-8582, Japan; Department of Neurosurgery, Graduate School of Medical Sciences, Kyushu University, 3-1-1 Maidashi, Higashi-ku, Fukuoka 812-8582, Japan; Department of Neurosurgery, Graduate School of Medical Sciences, Kyushu University, 3-1-1 Maidashi, Higashi-ku, Fukuoka 812-8582, Japan; Department of Neurosurgery, Graduate School of Medical Sciences, Kyushu University, 3-1-1 Maidashi, Higashi-ku, Fukuoka 812-8582, Japan; Department of Neurosurgery, Graduate School of Medical Sciences, Kyushu University, 3-1-1 Maidashi, Higashi-ku, Fukuoka 812-8582, Japan; Department of Neurosurgery, Graduate School of Medical Sciences, Kyushu University, 3-1-1 Maidashi, Higashi-ku, Fukuoka 812-8582, Japan; Department of Pathology, Graduate School of Medical Sciences, Kyushu University, 3-1-1 Maidashi, Higashi-ku, Fukuoka 812-8582, Japan; Department of Neurosurgery, Graduate School of Medical Sciences, Kyushu University, 3-1-1 Maidashi, Higashi-ku, Fukuoka 812-8582, Japan; Department of Neurosurgery, Graduate School of Medical Sciences, Kyushu University, 3-1-1 Maidashi, Higashi-ku, Fukuoka 812-8582, Japan; Department of Neurosurgery, Graduate School of Medical Sciences, Kyushu University, 3-1-1 Maidashi, Higashi-ku, Fukuoka 812-8582, Japan

**Keywords:** cerebrospinal fluid, copy number alterations, glioma, liquid biopsy, multiplex ligation-dependent probe amplification

## Abstract

**Background:**

Copy number alterations (CNAs) are common in diffuse gliomas and have been shown to have diagnostic significance. While liquid biopsy for diffuse glioma has been widely investigated, techniques for detecting CNAs are currently limited to methods such as next-generation sequencing. Multiplex ligation-dependent probe amplification (MLPA) is an established method for copy number analysis in pre-specified loci. In this study, we investigated whether CNAs could be detected by MLPA using patients’ cerebrospinal fluid (CSF).

**Methods:**

Twenty-five cases of adult diffuse glioma with CNAs were selected. Cell-free DNA (cfDNA) was extracted from the CSF, and DNA sizes and concentrations were recorded. Twelve samples, which had appropriate DNA sizes and concentrations, were subsequently used for analysis.

**Results:**

MLPA could be successfully performed in all 12 cases, and the detected CNAs were concordant with those detected using tumor tissues. Cases with epidermal growth factor receptor (EGFR) amplification, combination of gain of chromosome 7 and loss of chromosome 10, platelet-derived growth factor receptor alpha amplification, cyclin-dependent kinase 4 amplification, and cyclin-dependent kinase inhibitor 2A (CDKN2A) homozygous deletion were clearly distinguished from those with normal copy numbers. Moreover, EGFR variant III was accurately detected based on CNA.

**Conclusions:**

Thus, our results demonstrate that copy number analysis can be successfully performed by MLPA of cfDNA extracted from the CSF of patients with diffuse glioma.

**Key Points**

- MLPA was used for cfDNA copy number analysis in CSF from patients with glioma.
- EGFR amplification, Ch 7+/10−, and CDKN2A homozygous deletion concorded with tumor.
- DNA concentration and size were proposed as criteria for MLPA using cfDNA.

**Importance of the Study**

Copy number alterations (CNAs) are common and diagnostically significant in diffuse gliomas. Liquid biopsy for diffuse glioma is clinically important in monitoring tumor progression; however, techniques for detecting CNAs are currently limited to methods such as next-generation sequencing in liquid biopsy. Multiplex ligation-dependent probe amplification (MLPA) is an established method for copy number analysis in pre-specified loci. In this study, we demonstrated that CNAs could be detected by MLPA using patients’ cerebrospinal fluid. EGFR amplification, Ch 7+/10−, and CDKN2A homozygous deletion were found to be associated with tumor tissue. Moreover, EGFR variant III was accurately detected based on the copy number of each exon of EGFR. MLPA is extremely versatile, requires simple procedures, and can be carried out wherever general PCR and DNA fragment analysis are possible. Therefore, MLPA is considered to align with liquid biopsy, which is generally used for repeated evaluation for real-time disease monitoring.

For patients with adult-type diffuse glioma, which is the most common type and accounts for 81% of malignant primary brain tumors,^1^ molecular characterization is crucial for optimal diagnosis and treatment.^2,3^ Conventionally, molecular diagnosis is performed using the tumor tissue obtained by surgical resection; however, this can be challenging in cases where the surgical approach is difficult or has a higher risk of complications, such as for tumors located in the brainstem, thalamus, or eloquent areas. Moreover, after initial treatment involving surgical resection, radiation therapy, and chemotherapy, tumor recurrence is usually evaluated based on diagnostic imaging, because repeating surgery is highly invasive. However, the sensitivity of detection of tumor recurrence is limited, because it is not detectable unless the lesion grows.^4^ The concept of “liquid biopsy” or biopsy using patient body fluids, such as blood or cerebrospinal fluid (CSF), is expected to help overcome these problems. DNA floating in body fluid is called cell-free DNA (cfDNA); in particular, tumor-derived cfDNA is distinguished as cell-free tumor DNA (ctDNA) and is a target of liquid biopsy. We analyzed ctDNA in CSF using digital polymerase chain reaction (PCR) and established a method for detecting isocitrate dehydrogenase (IDH) 1 and promoter of telomerase reverse transcriptase (pTERT) mutations.^5^ This method is a cost-effective, highly sensitive approach to digital PCR and has a short reaction time, but it can detect a limited number of mutations.^6–9^ Multiplex ligation-dependent probe amplification (MLPA) is a relatively simple semi-quantitative PCR-based assay that can detect changes in DNA copy number at up to 50 loci. In the recent 2021 World Health Organization classification, the presence of epidermal growth factor receptor (EGFR) amplification or the combination of gain of chromosome 7 and loss of chromosome 10 (Ch 7+/10−) in adult IDH-wildtype astrocytoma and cyclin-dependent kinase inhibitor 2A (CDKN2A) homozygous deletion in IDH-mutant astrocytoma are designated with the highest malignant grade (grade 4) regardless of the histological malignancy.^3^ All of these molecular features can be identified by using the MLPA method,^10^ but to date, there has been no report of liquid biopsy for diffuse glioma using MLPA. In this study, we performed molecular diagnosis by MLPA using ctDNA derived from CSF for the first time.

## Materials and Methods

### Patients

**Figure 1.**
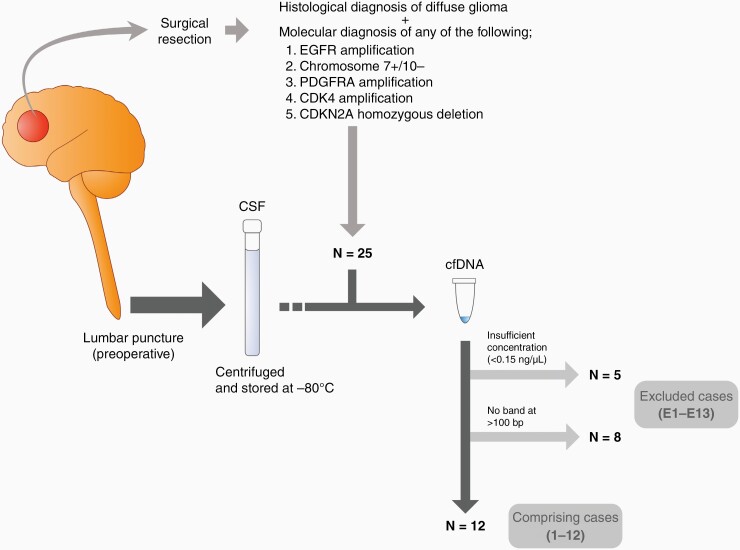
Flowchart indicating the inclusion and exclusion criteria used in this study. CDK4: cyclin-dependent kinase 4, CDKN2A: cyclin-dependent kinase inhibitor 2A, Ch 7+/10−: the combination of gain of chromosome 7 and loss of chromosome 10, CSF: cerebrospinal fluid, cfDNA: cell-free DNA, EGFR: epidermal growth factor receptor, PDGFRA: platelet-derived growth factor receptor alpha.

Twenty-five adult patients (aged >18 years) with newly diagnosed adult glioma with any copy number alterations (CNAs) for EGFR, phosphatase and tensin homolog (PTEN), CDKN2A, cyclin-dependent kinase 4 (CDK4), platelet-derived growth factor receptor alpha (PDGFRA), and from whom CSF was collected preoperatively between September 2019 to May 2022, were enrolled in this study. CNAs were evaluated using MLPA as described previously^11^ (Figure 1). The sample size was not determined statistically prior to the study, and the samples were not randomized. Tumor and CSF samples were given different sample numbers, and CNA analysis was blinded.

This study was approved by the local ethics committee (Kyushu University Institutional Review Board for Clinical Research; ethics review number: 30-332). All procedures performed were in accordance with the 1964 declaration of Helsinki (as revised in Fortaleza, Brazil, October 2013). All patients provided written informed consent.

### CSF Collection and cfDNA Extraction

CSF collected by lumbar puncture was centrifuged at 5000 × g for 10 min to remove residual cells and, then, stored at −80°C within 1 h, as described previously.^5^ cfDNA was extracted from 1 to 3 mL CSF using the QIAamp Circulating Nucleic Acid kit (55114; Qiagen Science, Germantown, ML, USA) according to the manufacturer’s instructions and eluted in a final volume of 20–60 µL (20 µL per 1 mL CSF). The extracted cfDNA samples were stored at 4°C and analyzed within 1 week.

### Plasma cfDNA Extraction

For supplementary experiments, plasma was separated from the blood of five healthy volunteers, and cfDNA was extracted by the same procedure. Plasma was separated by centrifuging the blood at 800 × g for 10 min to remove the cellular components, and the supernatant was centrifuged at 5000 × g for 10 min to pellet down residual cells. A 20 µL DNA sample was obtained from 1 mL of serum using the QIAamp Circulating Nucleic Acid kit (55114; Qiagen Science).

### Blood and tumor DNA extraction

Tumor DNA was extracted from snap-frozen (−80°C) intraoperatively obtained tumor samples using the QIAamp DNA mini kit (51304; Qiagen Science) according to the manufacturer’s protocol. Blood DNA was extracted using the QIAamp DNA Blood Maxi kit (51194; Qiagen Science). Patient blood was used for PCR-based loss of heterozygosity (LOH) analysis, and blood from healthy volunteers were used as a reference for comparative MLPA analysis. The reference DNA was also used in the experiment to confirm the lowest DNA concentration required for the MLPA reaction. Tris-EDTA buffer was used to adjust the DNA concentration from 0.050 ng/µL to 1 ng/µL in a stepwise manner.

### DNA Sample Quality Check

Quantification of cfDNA was performed using the Qubit 2.0 Fluorometer and the Qubit dsDNA HS Assay kit (Q32851; Thermo Fisher Scientific, Foster City, CA, USA). Electrophoresis was performed using the 4150 TapeStation system (Agilent Technologies, Inc., Santa Clara, CA, USA) and High Sensitivity D1000 ScreenTape to assess whether the cfDNA in the extracted DNA sample was appropriate for subsequent reactions. The corresponding data analysis were performed using the TapeStation software (Version: 4.1.0.1488, Agilent).

### MLPA

The copy numbers of the EGFR, PTEN, CDKN2A, PDGFRA, and CDK4 genes were evaluated using a commercial MLPA kit (P105-D3; MRC-Holland, Amsterdam, Netherlands) containing specific probes and 13 reference probes (SALSA® MLPA® Probemix P105-D3 Glioma-2; MRC-Holland). MLPA was performed according to the manufacturer’s protocol. Briefly, DNA was denatured at 98*°*C for 5 min, hybridized with the probe mix at 95°C for 1 min, and then incubated at 60*°*C for 16−20 h. Subsequently, ligation was performed at 54*°*C for 15 min, followed by a ligase inactivation step. Then, PCR was performed using the SALSA PCR primer mix and SALSA polymerase (EK1-FAM; MRC-Holland) (35 cycles; 95°C for 30 s, 60°C for 30 s, and 72°C for 1 min, with a final step at 72°C for 20 min). Denatured fragments were separated and quantified by electrophoresis using an ABI 3730 capillary sequencer (Applied Biosystems, Waltham, MA, USA) and analyzed using the GeneMapper® (Applied Biosystem) and Coffalyser® (MRC-Holland) software. Based on previous studies, the normal copy number thresholds were set at 1.2 and 0.8 for the detection of gains and losses, respectively, and ratios above 2.0 and below 0.4 were considered amplifications and homozygous deletions, respectively.^10,11^ As cfDNA concentration in the CSF is less than the DNA concentration recommended by the manufacturer, the minimum concentration for normal copy number analysis was confirmed experimentally. The blood DNA of healthy volunteers used as a reference for MLPA analysis was diluted, and MLPA was performed. MLPA quality was assessed based on the fragment MLPA reaction score and signal quality score, which were obtained using Coffalyser (Coffalyser.Net Reference Manual Version 02—For Coffalyser.Net v. 220513.1739. MRC-Holland).

### Conventional Genetic Analyses of Tumor‑tissue DNA

QIAamp DNA Mini kits (Qiagen Science) were used to isolate and purify DNA from snap-frozen (−80°C) intraoperative tumor samples obtained from all patients whose CSF was analyzed. The presence of IDH 1, IDH 2, pTERT C228T or C250T, and H3 K27M point mutations were confirmed by high-resolution melt analysis and subsequent Sanger sequencing, as described previously.^12–15^ LOH on chromosomes 1p and 19q was confirmed by a PCR-based LOH assay using microsatellite markers, as described previously.^15–18^

### pTERT Mutation Analysis Using Digital PCR

To examine the sensitivity of MLPA, pTERT mutation analysis was performed for cases positive for pTERT mutation by digital PCR as a supplementary experiment. Digital PCR was performed using the QuantStudio® 3D Digital PCR System (Life Technologies, Carlsbad, CA, USA). A commercial assay for pTERT (Assay ID: Hs000000092_rm and Hs000000093_rm, Life Technologies) containing primers and probes was used for detecting pTERT mutation of C228T and C250T. The final 14.5-μL digital PCR mixture was loaded onto a QuantStudio® 3D Digital PCR chip version 2 and subjected to PCR amplification using the QuantStudio® 3D GeneAmp PCR system 9700 with initial denaturing at 96°C for 10 min followed by 54 cycles of 55°C for 2 min and 98°C for 30 s, with a final step at 55°C for 2 min. Following the reaction, data were analyzed using QuantStudio® 3D Analysis Suite (version 3.1.6-PRC-build18) as previously reported.^5^ DNA samples from the corresponding tumor tissue were diluted to 3.3 ng/µL with Tris-EDTA buffer and used as positive controls.

### Neuroimaging Findings

For every patient, fluid-attenuated inversion-recovery and Gd-T1-weighted imaging scans were obtained preoperatively at our institution using a 1.5- or 3-T scanner and evaluated. Tumors were classified into 2 groups according to whether or not contrast enhancement was observed. The relationship with the lateral ventricles was classified based on the location of the contrast-enhanced tumor and contact with the subventricular zone.^19–21^

### Statistical Analysis

All statistical analyses were performed using JMP Pro version 16.0.0 (SAS Institute Inc., Cary, NC, USA). The level of statistical significance was set at *P* < .05. Clinical and molecular characteristics were evaluated using the chi-squared, Mann–Whitney U, and Fisher’s exact tests. In the experiment to determine the minimum DNA concentration required for MLPA using diluted DNA, the average fragment MLPA reaction score and signal quality score at each concentration were compared. The threshold was tested using Student’s *t*-test.

## Results

### Confirmation of Minimum Concentration Threshold and Quality Criteria of cfDNA for MLPA

Table 1 shows the clinical and molecular characteristics of the 25 cases included in this study. The median concentration of extracted cfDNA was 0.213 ng/µL (range, 0.075–1.355 ng/µL), which was much lower than what is recommended for an MLPA reaction (10 ng/µL, 50 ng); therefore, the minimum concentration of DNA that could be used in an MLPA reaction was determined.

**Table 1.** Clinical and Molecular Feature

Variable assessed	All cases (*n* = 25)	Sufficient quality (*n* = 12)	Insufficient quality (*n* = 13)	*P* value
Patient age, median (range)	59 (29−84)	59.5 (29−74)	58.5 (33−84)	.5859
Male sex (%)	15 (60)	8 (66.7)	7 (53.8)	.6882
Tumor size [mm], median (range)	54 (25−78)	55 (25−78)	37 (25−66)	.0384^*a*^
Contact with lateral ventricles (%)	20 (80)	11 (91.7)	9 (69.2)	.1857
Contrast-enhancement (%)	22 (88)	11 (91.7)	11 (84.6)	.5313
Radiological dissemination (%)	4 (16)	4 (33.3)	0 (0)	.0391^*a*^
IDH mutant (%)	2 (8.3)	1 (8.3)	1 (7.7)	.7400
TERT mutant (%)	15 (60)	9 (75)	6 (46.1)	.1442
1p/19q codeletion (%)	0 (0)	0 (0)	0 (0)	−
MGMT promoter methylation (%)	12 (48)	4 (33.3)	8 (61.5)	.2377
Copy number alteration (%)				
EGFR amplification	7 (28)	4 (33.3)	3 (23.1)	.6366
Ch 7+/10− (EGFR +/PTEN−)	14 (58.3)	9 (75)	5 (41.7)	.1069
PDGFRA amplification	2 (8)	1 (8.3)	1 (7.7)	1.0000
CDK4 amplification	1 (4)	1 (8.3)	0 (0)	.4800
CDKN2A homozygous deletion	18 (69.2)	9 (75)	9 (64.3)	.5503
ctDNA concentration [ng/µL], median (range)	0.213 (0.075−1.355)	0.421 (0.15−1.355)	0.182 (0.075−0.619)	.0167^*a*^

^*a*^ indicates statistical significance (*P* < .05).

Abbreviations: CDK4: cyclin-dependent kinase 4, CDKN2A: cyclin-dependent kinase inhibitor 2A, Ch 7+/10−: the combination of gain of chromosome 7 and loss of chromosome 10, ctDNA: cell-free tumor DNA, EGFR: epidermal growth factor receptor, IDH: isocitrate dehydrogenase, MGMT: O(6)-methylguanine-DNA methyltransferase, PDGFRA: platelet-derived growth factor receptor alpha, PTEN: phosphatase and tensin homolog, TERT telomerase reverse transcriptase.

**Figure 2.**
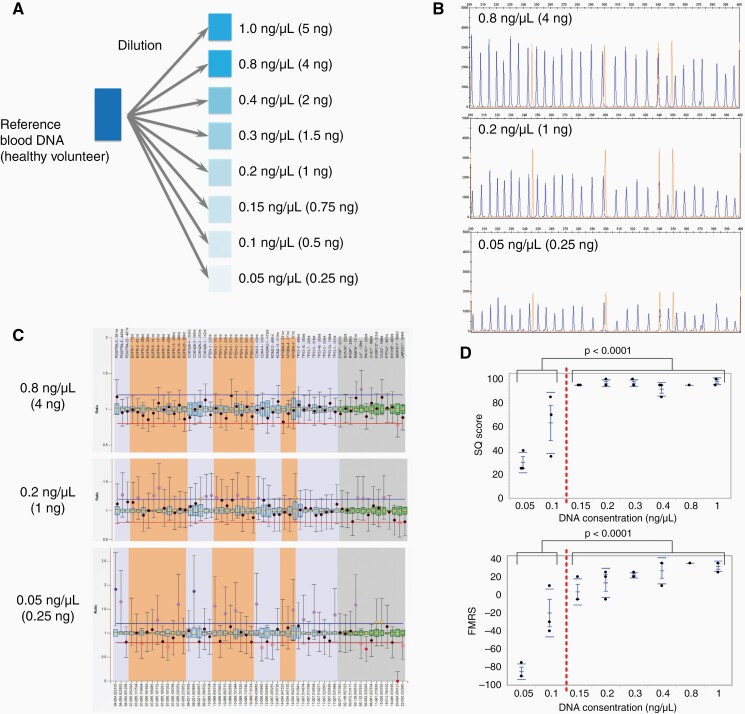
Exploratory experiment to determine the minimum DNA concentration threshold for MLPA reactions. (A) Stepwise dilution of normal copy number DNA samples containing 0.05–1.0 ng/µL genomic DNA. Examples of MLPA results obtained using assay P105—peak electrophoresis (B) and analyzed ratio chart (C) patterns are depicted. Peak electrophoresis signals decrease as the concentration decreases, and the 95% confidence interval on the ratio chart is also extended. (D) The signal quality score and fragment MLPA reaction score, which indicate MLPA quality, deteriorated significantly below 0.15 ng/µL. MLPA: multiplex ligation-dependent probe amplification, SQ: signal quality, FRRS: fragment MLPA reaction score.

Blood DNA of a healthy volunteer with a normal copy number, which was used as a reference for MLPA analysis, was diluted, and an MLPA experiment was performed (Figure 2A–D). Based on fragment analysis using GeneMapper, it was confirmed that amplification by PCR was obtained even with a minimum sample concentration of 0.05 ng/µL (Figure 2B). It was erroneously determined that the copy number in the low-concentration sample was not the normal copy number on the Coffalyser comparative analysis ratio chart, and the 95% confidence interval of each probe was extremely expanded (Figure 2C). The fragment MLPA reaction score and signal quality scores obtained using Coffalyser were obviously maintained in samples with concentration above 0.15 ng/µL (*P* < .0001, Figure 2D), and normal copy number was analyzed correctly. Therefore, the cutoff cfDNA concentration for the MLPA reaction was set to 0.15 ng/µL. Five samples with cfDNA concentration <0.15 ng/µL were excluded from the MLPA analysis.

Additionally, nine samples containing only highly digested DNA fragments smaller than 100 bp in size were excluded, because tumor-derived 150 bp and 300 bp DNA is abundant in CSF,^5,22^ and the MLPA primer set published by the manufacturer is designed to hybridize to DNA ≥100 bp in size (Figure 1).

### Copy Number Analysis Using cfDNA From CSF by MLPA

MLPA reactions and copy number analysis were successfully performed for all 12 of the 25 cases that satisfied the criteria for MLPA (Figure 1). Table 1 shows a comparison of cases that passed the quality check and those that did not. Cases that passed the quality check had a larger tumor diameter and a higher frequency of dissemination. Ch 7+/10− was also slightly more common in such cases than in cases with unsatisfactory quality. Supplementally, we tried additional MLPA experiments for 13 cases that did not pass the quality check (Supplementary Table S1); none of them gave meaningful results. In 10 cases, the probe did not successfully hybridize, and the subsequent PCR did not result in amplification of the target fragment. There were reactions of some probes in 2 cases, but these were insufficient for copy number evaluation. Although it was possible to analyze the copy numbers for most probes in one case, the electrophoresis signals, including those for the internal control probe, were low and unreliable for copy number determination and for comparison with the tumor.

**Figure 3.**
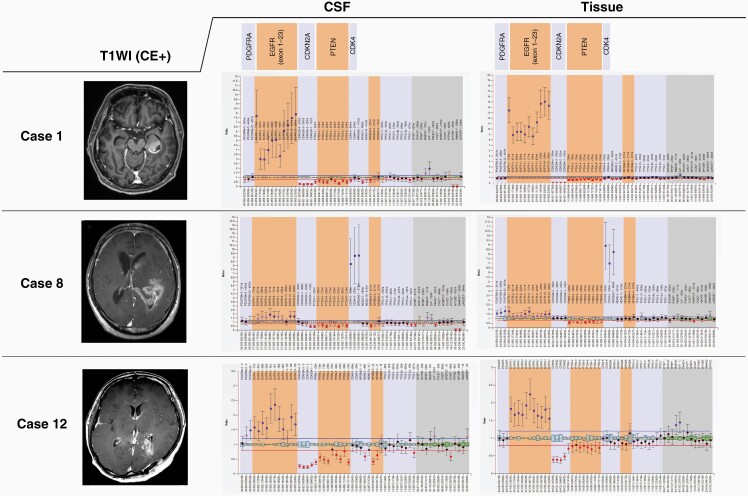
Examples of representative copy number analysis MLPA results obtained using assay P105. Ratio charts indicate relative alterations from normal PDGFRA, EGFR, CDKN2A, PTEN, and CDK4 copy numbers in that order from the left. Copy number alterations detected by MLPA using cell-free DNA from cerebrospinal fluid (left) were concordant with those detected using tumor tissue (right). Contrast-enhanced T1-weighted images of each case are also shown. CDK4: cyclin-dependent kinase 4, CDKN2A: cyclin-dependent kinase inhibitor 2A, EGFR: epidermal growth factor receptor, MLPA: multiplex ligation-dependent probe amplification, PDGFRA: platelet-derived growth factor receptor alpha, PTEN: phosphatase and tensin homolog.

Details of the 12 cases are shown in Table 2. Figure 3 shows examples of comparisons between the results of MLPA analysis using the CSF-derived cfDNA and using tumor tissue. The deviations were larger and the error bars were longer for all the probes, including 13 internal controls (gray background at the right end of the ratio chart), but this did not affect the assessment of the copy number. The results obtained using cfDNA were concordant with those for the tumor tissue. Moreover, case 1 reflected the amplification of EGFR and the result of variant III. It was also possible to evaluate difference in deletion, as PTEN showed heterozygous deletion and CDKN2A showed homozygous deletion in Cases 1 and 12.

**Table 2.** Background and Result of MLPA Using ctDNA From Cerebrospinal Fluid

Case	Age	Sex	Integrated diagnosis (grade)	Histology	ctDNA condition		Radiology				Molecular diagnosis (tumor tissue)							
					Conc. (ng/µL)	>100 bp	CE	Size (mm)	LV-contact	Dissemination	IDH	MGMT	TERT	EGFR	Ch 7+/10−	PDGFRA	CDK4	CDKN2A
1	74	M	GBM, IDH-wt (4)	GBM	0.574	+	+	25	+	−	Wt	u	C228T	Amp (vIII)	+	Wt	Wt	Homo
2	53	M	GBM, IDH-wt (4)	GBM	1.105	+	+	77	+	+	Wt	u	C228T	Amp	+	Wt	Wt	Homo
3	44	F	Astrocytoma, IDH-mut (4)	AA	0.186	+	−	53	−	−	R132H	m	Wt	Amp	-−	Wt	Wt	Wt
4	64	M	GBM, IDH-wt (4)	GBM	0.150	+	+	71	+	−	Wt	u	C228T	Gain	+	amp	Wt	Homo
5	33	F	GBM, IDH-wt (4)	GBM	0.530	+	+	54	+	−	Wt	m	C228T	Wt	−	Wt	Wt	Homo
6	61	F	GBM, IDH-wt (4)	GBM	1.355	+	+	55	+	−	Wt	u	Wt	Gain	+	Wt	Wt	Wt
7	29	M	GBM, IDH-wt (4)	AA	0.464	+	+	73	+	+	Wt	u	C228T	Wt	−	Wt	Wt	Homo
8	66	M	GBM, IDH-wt (4)	GBM	0.173	+	+	78	+	+	Wt	u	Wt	Gain	+	gain	amp	Wt
9	68	M	GBM, IDH-wt (4)	GBM	0.213	+	+	38	+	−	Wt	m	C250T	Gain	+	Wt	Wt	Homo
10	62	F	GBM, IDH-wt (4)	GBM	0.445	+	+	55	+	−	Wt	m	C228T	Amp	+	Wt	Wt	Homo
11	51	M	GBM, IDH-wt (4)	GBM	0.303	+	+	60	+	+	Wt	u	C250T	Gain	+	Wt	Wt	Homo
12	58	M	GBM, IDH-wt (4)	GBM	0.397	+	+	50	+	−	Wt	u	C228T	Gain	+	Wt	Wt	Homo
Case	MLPA (CSF)																	
	EGFR (M/U)			CCh 7+/10–(M/U)		PDGFRA (M/U)				CDK4 (M/U)				CDKN2A (M/U)				
1	Amp (vIII)		(M)	+	(M)	Wt		(M)		Wt		(M)		Homo		(M)		
2	Gain		(U)	+	(M)	Wt		(M)		Wt		(M)		Hetero		(U)		
3	Amp		(M)	−	(M)	Wt		(M)		Wt		(M)		Ni		(U)		
4	Gain		(M)	+	(M)	Amp		(M)		Hetero		(U)		Hetero		(U)		
5	Wt		(M)	−	(M)	Wt		(M)		Wt		(M)		Homo		(M)		
6	Gain		(M)	+	(M)	Wt		(M)		Wt		(M)		Hetero		(U)		
7	Wt		(M)	−	(M)	Wt		(M)		Wt		(M)		Homo		(M)		
8	Gain		(M)	+	(M)	Wt		(U)		Amp		(M)		Wt		(M)		
9	Gain		(M)	+	(M)	Wt		(M)		Wt		(M)		Homo		(M)		
10	Amp		(M)	+	(M)	Wt		(M)		Wt		(M)		Homo		(M)		
11	Wt		(U)	−	(U)	Wt		(M)		Wt		(M)		Hetero		(U)		
12	Gain		(M)	+	(M)	Wt		(M)		Wt		(M)		Homo		(M)		

Abbreviations: Amp: amplification, CDK4: cyclin-dependent kinase 4, CDKN2A: cyclin-dependent kinase inhibitor 2A, CE: contrast-enhancement, Ch 7+/10−: the combination of gain of chromosome 7 and loss of chromosome 10, Conc.: concentration, CSF: cerebrospinal fluid, ctDNA: cell-free tumor DNA, EGFR: epidermal growth factor receptor, GBM: Glioblastoma, Hetero: heterozygous deletion, Homo: homozygous deletion, IDH: isocitrate dehydrogenase, LV-contact: contact with lateral ventricles, m: methylated, MGMT: O(6)-methylguanine-DNA methyltransferase, MLPA: Multiplex ligation-dependent probe amplification, Ni: not informative, PDGFRA: platelet-derived growth factor receptor alpha, TERT telomerase reverse transcriptase, u: unmethylated, vIII: variant type III, (M): matched, (U): unmatched.

In contrast, there was one case in which EGFR amplification was determined to be gain and four cases in which CDKN2A homozygous deletion was determined to be heterozygous deletion. Regarding the detection accuracy of MLPA, the sensitivity of PDGFRA amplification and CDK4 amplification was 100% (one out of one), Chr 7+/10− was 88.9% (8 out of 9 cases), and EGFR amplification and CDKN2A homozygous deletion were 75.0% (three out of four cases) and 60% (six out of 10 cases), respectively. The specificities were all 100%.

To confirm that the CNAs were not detected incidentally, additional copy number analysis with MLPA using cfDNA from plasma of 15 samples of 5 healthy volunteers (Supplementary Table S2) was performed. All samples were evaluated for the concentration and size of the DNA, and they met the criteria. As shown in Supplementary Figure S1, EGFR, PTEN, CDKN2A, and CDK4 were all assessed as normal copy numbers.

### Supplementary Experiment to Assess the Sensitivity of MLPA

To further evaluate the sensitivity of MLPA, pTERT mutation analysis was performed for the cases possessing a pTERT mutation. Digital PCR was performed on 14 of 15 cases with a pTERT mutation, and one case had an insufficient volume of DNA sample for the experiment (case 4). As described above, MLPA was successful in 12 of 25 patients (48% sensitivity); digital PCR, on the other hand, successfully revealed the corresponding mutation detected in the tumor tissue in 13 of 14 patients (92.9%) (Supplementary Table S3). It should be noted that digital PCR was able to detect pTERT mutations in five out of six samples for which the MLPA reaction was unsuccessful.

## Discussion

In this study, liquid biopsy using the MLPA method was performed for successful copy number analysis of cfDNA derived from CSF. MLPA is an established method for copy number analysis in cancer research, including cases of glioma.^11,23–26^ However, to the best of our knowledge, there are no reports of using MLPA for liquid biopsy for diffuse glioma. This study is the first to report that EGFR amplification, Ch 7+/10−, PDGFRA amplification, CDK4 amplification, and CDKN2A homozygous deletion, which are important for glioma molecular diagnosis, can be analyzed using cfDNA derived from CSF and MLPA if certain conditions regarding the sample cfDNA are met.

Liquid biopsy of brain tumors has been actively studied with the ultimate aim of developing a minimally invasive approach for diagnosis and disease monitoring.^5,8,22,27–37^ In particular, there are many reports using digital PCR, which is recognized as a promising tool with high sensitivity for analysis of samples with low ctDNA concentration.^5,8,29,33,35^ However, even though it is a powerful technique for the detection of a limited number of tumor driver mutations, owing to its underlying principle, digital PCR has a practical limitation in the clinical situations, where multiple genetic diagnoses are required, including copy number analysis, and where the obtained samples are limited. Conversely, it is possible to evaluate up to 50 loci simultaneously in one MLPA reaction using a small amount of DNA.^38^ Furthermore, as it is a semi-quantitative technique, MLPA also enables the evaluation of CNAs. The relative copy number quantification according to the comparison with the control is from the magnitude of the alterations and not just a binary diagnosis of mutant or wild-type status; the copy number can also be categorized as amplification, gain, heterozygous deletion, or homozygous deletion. Moreover, the rate of amplification makes it possible to diagnose EGFR variants. In fact, the CNAs detected by applying MLPA analysis to cfDNA extracted from CSF in this study were concordant with those in the corresponding tumor tissues. These CNAs are considered to reflect tumor-specific changes rather than accidental coincidence because normal copy number was also analyzed in MLPA of cfDNA derived from plasma of healthy volunteers as demonstrated by the supplementary experiment. Whole exome sequencing or shallow whole genome sequencing with next-generation sequencing is considered as a promising method for liquid biopsy.^28,34,36^ Innovations in next-generation sequencing technology could possibly enable comprehensive molecular diagnosis; however, there are several major obstacles in the clinical settings, including cost and limited accessibility. In that respect, MLPA is extremely versatile, requires simple procedures, and can be carried out wherever general PCR and DNA fragment analysis are possible. Therefore, this highly portable method is considered to align with liquid biopsy, which is generally used for repeated evaluation for real-time disease monitoring.

It should be noted that the ratio of ctDNA to normal-tissue-derived cfDNA is greatly affected in the copy number analysis. cfDNA in body fluid naturally contains ctDNA derived from apoptotic tumor cells and other cfDNA derived from non-tumor cells. Moreover, surgically resected tumors contain certain amounts of necrotic brain cells and inflammatory cells. Especially when blood is used instead of CSF, it is expected that germline DNA from leukocytes, which are present in abundance, would result in extreme dilution of ctDNA. Previous studies have reported that ctDNA was clearly detectable and that the tumor-derived to non-tumor-derived ratio was predominantly higher for CSF than for plasma.^22,33^

MLPA is a useful technique; however, it has rarely been used in the context of liquid biopsy until now.^39^ There was uncertainty regarding whether MLPA reactions could be performed correctly using cfDNA, which is highly digestible at low concentrations. In this study, cases, in which MLPA was feasible were selected based on 2 criteria: cfDNA sample concentration and molecular weight. The concentration of cfDNA extracted from CSF was too low when compared to that recommended for MLPA by the manufacturer (at least 4 ng/µL).^40^ However, MLPA of diluted normal copy number samples showed that 0.15 ng/µL was sufficient for normal copy number determination. Thus, the threshold concentration for MLPA reactions was set at ≥0.15 ng/µL. It should be noted that this is a criterion established in this study to ensure CNA quantification. As all reactions, including hybridization, ligation, and subsequent PCR using each probe, were successfully performed at the lowest DNA concentration of 0.05 ng/µL, it may be acceptable to lower the cutoff in the situation where the quantification of copy number is allowed to be sacrificed, and a binary result of whether a gene mutation is present or absent is acceptable. In addition to the concentration, the qualitative criterion of whether the sample includes DNA size ≥100 bp was set. This is because the hybridization and ligation of specific probes are, in principle, impossible in samples that have only digested DNA <100 bp in size. In fact, supplementary experiments showed that the MLPA reaction was not successful in cases without the necessary DNA bands. There are several reports on the size of ctDNA in CSF, and it is known that the ctDNA is detected at approximately 150 and 300 bp, especially in a situation, where cell apoptosis is actively occurring in malignant tumors.^5,22^ The requirement of confirmation of DNA of size ≥100 bp may not only be a limitation of MLPA in principle, but could also result in the selection of cases, where apoptosis is active and undigested ctDNA is abundant.

In this study, MLPA and copy number analysis were successfully performed using cfDNA in cases that fulfilled these 2 criteria, and conversely, MLPA reactions and subsequent analysis failed in cases that did not fulfill them. Therefore, it is considered that the 2 criteria have a certain implication for detection of cases, which are suitable for MLPA. In contrast, it remains unclear how many cases meet the 2 criteria in all glioma cases. Previous studies have revealed that ctDNA is present in the CSF in cases of metastatic brain tumors and high-grade glioma but is less likely to be detected in lower-grade glioma cases.^28,35,36,41^ It was also reported that contact with CSF reservoir in cases of leptomeningeal disease made ctDNA detection easier.^41^ In our series, cases with radiological dissemination or a larger tumor size met the criteria for MLPA. However, case 3 (IDH-mutant anaplastic astrocytoma with neither dissemination nor contact with lateral ventricles) met the criteria, and CNA was successfully analyzed. Further exploration is required to reveal the factors that make it possible for CNA to be analyzed using MLPA.

One of the crucial issues in the future is the sensitivity of MLPA. In the present study, MLPA was successful in 12 of 25 cases, which represents a sensitivity of 48%. In contrast, digital PCR had a higher sensitivity (92.9%) to detect pTERT mutations and even detected the mutation in 5 of 6 cases for which the MLPA reaction was unsuccessful (Supplementary Table S3). This shows that the detection ability of MLPA is inferior to that of digital PCR, which has been widely used for liquid biopsy, and indicates that ctDNA was certainly present in samples for which MLPA was unsuccessful. To increase the sensitivity of MLPA, not only improving the MLPA protocol but also identifying ways to obtain high-quality cfDNA samples will be important. In particular, a previous report showed that the amount of cfDNA in CSF reduced to one-third in 2 h,^42^ indicating a short half-life of cfDNA. Nine of the 13 cases excluded from the MLPA were revealed to have highly digested DNA in quality check although they had a certain concentration. This may suggest the possibility that digestion occurred during the process from collection to storage of CSF. Remediation of procedures to obtain high-quality cfDNA from CSF may lead to improved MLPA sensitivity.

This study had several limitations. One of the major limitations is the small sample size and limited cases of tumors with CNA. Cases with normal copy numbers were excluded, and as a result, lower-grade gliomas were not included. The criteria for defining CNA in this investigation were set at the same thresholds as those for conventional MLPA for tumor tissue; however, these thresholds may need more consideration. ctDNA is naturally diluted by normal copy number cfDNA derived from normal cells and, therefore, the detected copy number may be biased toward normal copy number. Further investigation with larger samples and more lenient inclusion criteria is needed to further clarify these aspects.

## Conclusions

Copy number analysis of adult diffuse glioma with CNAs was successfully performed using MLPA of ctDNA extracted from patients’ CSF. This approach can be expected to facilitate further development of the liquid biopsy approach for diagnosis and clinical management, especially in the context of real-time monitoring of glioma progression.

## Supplementary Material

vdac178_suppl_Supplementary_Figure_S1

vdac178_suppl_Supplementary_Figure_S1

vdac178_suppl_Supplementary_Table_S1

vdac178_suppl_Supplementary_Table_S2

vdac178_suppl_Supplementary_Table_S3

vdac178_suppl_Supplementary_Data
