# Supplementary figures and images for: Liquid biopsy with multiplex ligation-dependent probe amplification targeting cell-free tumor DNA in cerebrospinal fluid from patients with adult diffuse glioma
Source: Neurooncol Adv. 2022 Nov 25;5(1):vdac178. doi: 10.1093/noajnl/vdac178 (PMC9977236; doi:10.1093/noajnl/vdac178)

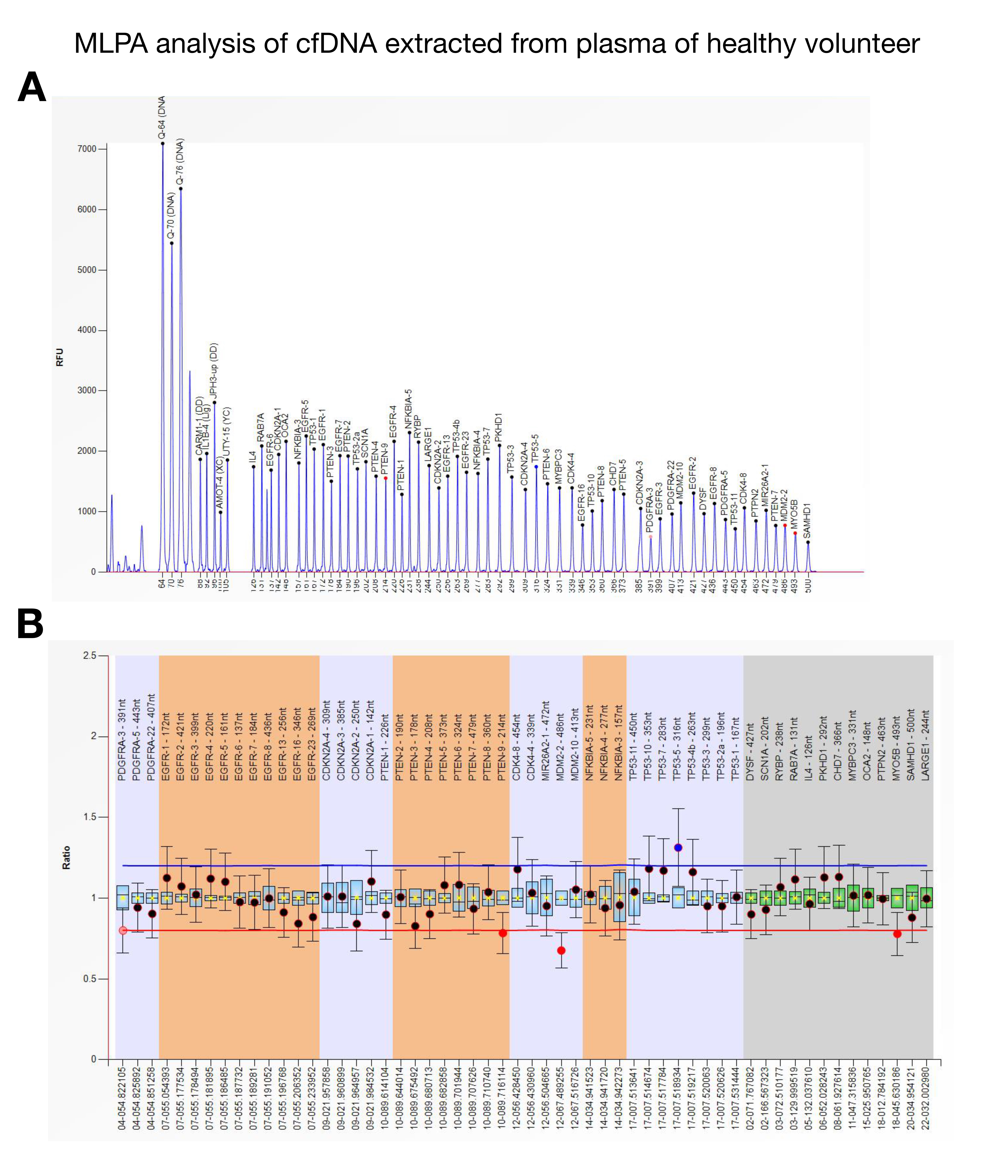

Supplement: vdac178_suppl_Supplementary_Figure_S1 [file vdac178_suppl_supplementary_figure_s1.jpeg]
