# Supplementary material for: Liquid biopsy with multiplex ligation-dependent probe amplification targeting cell-free tumor DNA in cerebrospinal fluid from patients with adult diffuse glioma
Source: Neurooncol Adv. 2022 Nov 25;5(1):vdac178. doi: 10.1093/noajnl/vdac178 (PMC9977236; doi:10.1093/noajnl/vdac178)
Supplement: vdac178_suppl_Supplementary_Table_S1 [file vdac178_suppl_supplementary_table_s1.docx]

**Table S1. Background and result of MLPA using cfDNA from cerebrospinal fluid**

| Case | Age | Sex | Integrated diagnosis  (grade) | Histology | cfDNA condition | | Radiology | | | | Molecular diagnosis (tumor tissue) | | | | | | | |
| --- | --- | --- | --- | --- | --- | --- | --- | --- | --- | --- | --- | --- | --- | --- | --- | --- | --- | --- |
|  |  |  |  |  | Conc.  (ng/µL) | >100bp | CE | Size  (mm) | LV-contact | Dissemination | IDH | MGMT | TERT | EGFR | Ch 7+/10- | PDGFRA | CDK4 | CDKN2A |
| E1 | 84 | F | GBM, IDH-wt (4) | GBM | 0.116 | - | + | 54 | + | - | Wt | u | Wt | Wt | - | amp | Wt | wt |
| E2 | 61 | F | GBM, IDH-wt (4) | GBM | 0.6185 | - | + | 50 | - | - | Wt | m | Wt | Wt | - | gain | Wt | wt |
| E3 | 61 | F | GBM, IDH-wt (4) | GBM | 0.402 | - | + | 55 | + | - | Wt | u | Wt | gain | + | Wt | Wt | wt |
| E4 | 54 | M | GBM, IDH-wt (4) | GBM | 0.113 | - | + | 33 | + | - | Wt | m | C228T | amp | + | Wt | Wt | homo |
| E5 | 58 | M | GBM, IDH-wt (4) | GBM | 0.1345 | - | + | 37 | - | - | Wt | m | C228T | amp | + | Wt | Wt | homo |
| E6 | 77 | F | Astrocytoma,  IDH-wt, NEC (3) | AA | 0.172 | - | - | 36 | + | - | Wt | u | Wt | Wt | - | Wt | Wt | homo |
| E7 | 44 | M | GBM, IDH-wt (4) | GBM | 0.3645 | - | + | 54 | + | - | Wt | u | C228T | Wt | - | Wt | Wt | homo |
| E8 | 46 | M | GBM, IDH-wt (4) | GBM | 0.075 | - | - | 66 | - | - | R132H | m | Wt | gain | + | Wt | Wt | homo |
| E9 | 76 | M | GBM, IDH-wt (4) | GBM | 0.182 | - | + | 25 | + | - | Wt | m | C250T | Wt | - | Wt | Wt | homo |
| E10 | 33 | F | Astrocytoma,  IDH-wt, NEC (3) | AA | 0.197 | - | + | 32 | + | - | Wt | m | Wt | Wt | - | Wt | Wt | homo |
| E11 | 51 | M | GBM, IDH-wt (4) | GBM | 0.1235 | - | + | 65 | + | - | Wt | m | C250T | gain | + | Wt | Wt | wt |
| E12 | 78 | F | GBM, IDH-wt (4) | GBM | 0.228 | - | + | 30 | + | - | Wt | m | Wt | Wt | - | Wt | Wt | homo |
| E13 | 59 | M | GBM, IDH-wt (4) | GBM | 0.209 | - | + | 28 | - | - | Wt | u | C228T | amp | + | Wt | Wt | homo |

| Case | MLPA (CSF) | | | | | | | | | |
| --- | --- | --- | --- | --- | --- | --- | --- | --- | --- | --- |
|  | EGFR (M/U) | | Ch 7+/10- (M/U) | | PDGFRA (M/U) | | CDK4 (M/U) | | CDKN2A (M/U) | |
| E1 | Ni | (U) | Ni | (U) | Gain | (U) | Wt | (M) | Ni | (U) |
| E2 | N/A | | N/A | | N/A | | N/A | | N/A | |
| E3 | N/A | | N/A | | N/A | | N/A | | N/A | |
| E4 | N/A | | N/A | | N/A | | N/A | | N/A | |
| E5 | Ni | (U) | Ni | (U) | Ni | (U) | Ni | (U) | Ni | (U) |
| E6 | N/A | | N/A | | N/A | | N/A | | N/A | |
| E7 | N/A | | N/A | | N/A | | N/A | | N/A | |
| E8 | N/A | | N/A | | N/A | | N/A | | N/A | |
| E9 | N/A | | N/A | | N/A | | N/A | | N/A | |
| E10 | N/A | | N/A | | N/A | | N/A | | N/A | |
| E11 | N/A | | N/A | | N/A | | N/A | | N/A | |
| E12 | Wt | (M) | - | (M) | Gain | (U) | Wt | (M) | Wt | (U) |
| E13 | N/A | | N/A | | N/A | | N/A | | N/A | |

Amp: amplification, CE: contrast-enhancement, cfDNA: cell-free DNA, Conc.: concentration, Homo: homozygous deletion, LV-contact: contact with lateral ventricles, m: methylated, N/A: not available (fragment analysis failure), Ni: not informative, u: unmethylated, vIII: variant type III, (M): matched, (U): unmatched
