# Supplementary material for: Liquid biopsy with multiplex ligation-dependent probe amplification targeting cell-free tumor DNA in cerebrospinal fluid from patients with adult diffuse glioma
Source: Neurooncol Adv. 2022 Nov 25;5(1):vdac178. doi: 10.1093/noajnl/vdac178 (PMC9977236; doi:10.1093/noajnl/vdac178)
Supplement: vdac178_suppl_Supplementary_Table_S2 [file vdac178_suppl_supplementary_table_s2.docx]

**Table S2. Result of MLPA using cfDNA of plasma from healthy volunteers**

|  |  | cfDNA condition | | MLPA | | | | |
| --- | --- | --- | --- | --- | --- | --- | --- | --- |
| Sample | Volunteer | Conc.  (ng/µL) | >100 bp | EGFR | Ch 7+/10- | PDGFRA | CDK4 | CDKN2A |
| P1 | A | 0.822 | + | Wt | - | Wt | Wt | Wt |
| P2 | A | 0.785 | + | Wt | - | Wt | Wt | Wt |
| P3 | B | 0.256 | + | Wt | - | Wt | Wt | Wt |
| P4 | B | 0.342 | + | Wt | - | Wt | Wt | Wt |
| P5 | B | 0.716 | + | Wt | - | Wt | Wt | Wt |
| P6 | C | 0.399 | + | Wt | - | Wt | Wt | Wt |
| P7 | C | 0.273 | + | Wt | - | Wt | Wt | Wt |
| P8 | D | 1.245 | + | Wt | - | Wt | Wt | Wt |
| P9 | D | 0.786 | + | Wt | - | Wt | Wt | Wt |
| P10 | D | 0.529 | + | Wt | - | Wt | Wt | Wt |
| P11 | E | 0.623 | + | Wt | - | Wt | Wt | Wt |
| P12 | E | 1.490 | + | Wt | - | Wt | Wt | Wt |
| P13 | E | 0.215 | + | Wt | - | Wt | Wt | Wt |
| P14 | E | 0.973 | + | Wt | - | Wt | Wt | Wt |
| P15 | E | 1.225 | + | Wt | - | Wt | Wt | Wt |

CDK4: cyclin dependent kinase 4, CDKN2A: cyclin dependent kinase inhibitor 2A, cfDNA: cell-free DNA, Ch 7+/10-: the combination of gain of chromosome 7 and loss of chromosome 10, Conc.: concentration, cfDNA: cell-free DNA, EGFR: epidermal growth factor receptor, PDGFRA: platelet derived growth factor receptor alpha
