# Supplementary material for: Liquid biopsy with multiplex ligation-dependent probe amplification targeting cell-free tumor DNA in cerebrospinal fluid from patients with adult diffuse glioma
Source: Neurooncol Adv. 2022 Nov 25;5(1):vdac178. doi: 10.1093/noajnl/vdac178 (PMC9977236; doi:10.1093/noajnl/vdac178)
Supplement: vdac178_suppl_Supplementary_Table_S3 [file vdac178_suppl_supplementary_table_s3.docx]

**Table S3. TERT promoter mutation analysis using digital PCR**

| Case | Successful CSF MLPA | TERT sequence | Digital PCR |
| --- | --- | --- | --- |
| 1 | + | C228T | C228T |
| 2 | + | C228T | C228T |
| 4 | + | C228T | N/A |
| 5 | + | C228T | C228T |
| 7 | + | C228T | C228T |
| 9 | + | C250T | C250T |
| 10 | + | C228T | C228T |
| 11 | + | C250T | C250T |
| 12 | + | C228T | C228T |
| E4 | - | C228T | C228T |
| E5 | - | C228T | C228T |
| E7 | - | C228T | C228T |
| E9 | - | C250T | C250T |
| E11 | - | C250T | C250T |
| E13 | - | C228T | WT * |

N/A: not available, WT: wild type

Asterisks shows false negative.
