# Supplementary material for: Liquid biopsy with multiplex ligation-dependent probe amplification targeting cell-free tumor DNA in cerebrospinal fluid from patients with adult diffuse glioma
Source: Neurooncol Adv. 2022 Nov 25;5(1):vdac178. doi: 10.1093/noajnl/vdac178 (PMC9977236; doi:10.1093/noajnl/vdac178)
Supplement: vdac178_suppl_Supplementary_Data [file vdac178_suppl_supplementary_data.docx]

**Supplementary Figure legends**

Figure S1

Example of copy number analysis of cell-free DNA extracted from plasma obtained from a healthy volunteer. MLPA results obtained using assay P105 are shown. Electropherogram shows that all probes are reacted (upper). Ratio chart indicates relative alterations from normal PDGFRA, EGFR, CDKN2A, PTEN, and CDK4 copy numbers in that order from the left (lower). All copy numbers at these loci were assessed as normal.

CDK4: cyclin dependent kinase 4, CDKN2A: cyclin dependent kinase inhibitor 2A, EGFR: epidermal growth factor receptor, MLPA: Multiplex ligation-dependent probe amplification, PDGFRA: platelet derived growth factor receptor alpha, PTEN: phosphatase and tensin homolog
